# Natural Interactions between *S. haematobium* and *S. guineensis* in the Republic of Benin

**DOI:** 10.1100/2012/793420

**Published:** 2012-05-03

**Authors:** Hélène Moné, Stéphanie Minguez, Moudachirou Ibikounlé, Jean-François Allienne, Achille Massougbodji, Gabriel Mouahid

**Affiliations:** ^1^Université de Perpignan Via Domitia, Ecologie et Evolution des Interactions, UMR 5244, 66860 Perpignan, France; ^2^CNRS, Ecologie et Evolution des Interactions, UMR 5244, 66860 Perpignan, France; ^3^Département de Zoologie et Génétique, Faculté des Sciences et Techniques, Université d'Abomey-Calavi, 01BP526 Cotonou, Benin; ^4^Laboratoire de Parasitologie-Mycologie, Faculté des Sciences de la Santé, Université d'Abomey-Calavi, 01BP188 Cotonou, Benin

## Abstract

Schistosomiasis is a parasitic disease which affects millions of people around the world, particularly in Africa. In this continent, different species are able to interbreed, like *Schistosoma haematobium* and *Schistosoma guineensis*, two schistosome species infecting humans. The Republic of Benin is known to harbor *S. haematobium*, but its geographical situation in between Nigeria, Mali, and Burkina Faso, where *S. guineensis* was found, raises the question about the possible presence of *S. haematobium/S. guineensis* hybrids in this country. We conducted morphological analyses on schistosome eggs and molecular analyses on schistosome larvae (high resolution melting (HRM) analysis and gene sequencing) in order to detect any natural interaction between these two species of schistosomes. The morphological results showed the presence of three egg morphotypes (*S. haematobium, S. guineensis*, and intermediate). Three genotypes were detected by ITS2 rDNA HRM analysis: *S. haematobium, S. guineensis*, and hybrid, and their percentages confirmed the results of the morphological analysis. However, sequencing of the CO1 mtDNA gene showed that all the samples from Benin belonged to *S. haematobium*. Our results provide the first evidence of introgression of *S. guineensis* genes in *S. haematobium* in Benin.

## 1. Introduction

Hybridization is a way for one organism to invade the genome of another; examples of limited invasions of genomes by this method are widespread and have important implications for evolutionary biology [1]. This mechanism may generate diversity through introgression of genes across species [2]. Then, hybridization can lead to disease-causing organisms acquiring novel genotypes, potentially expanding their geographical range and leading to novel ecological adaptations detrimental to human populations [3].

Schistosomes are a fascinating group of digenean trematodes because they are gonochoric, and species of the same genus that are sufficiently closely related may hybridize. These parasites are the agents of schistosomiasis, a disease affecting more than 200 million people worldwide, predominantly not only in Africa but also in South America and Asia [4]. The schistosome life cycle includes two obligatory hosts: (i) a homeothermic vertebrate (amongst which humans are included) within which the adult male and female parasites mate and reproduce, producing thousands of eggs in the venous system and (ii) a freshwater snail in which the parasite reproduces asexually, forming numerous sporocysts. The vertebrate-to-snail transmission is mediated by a swimming larval stage (the miracidium), which hatches from the egg. The snail-to-vertebrate transmission is mediated by cercariae, which are swimming larvae derived from sporocysts. Each larval transmission stage penetrates actively through its respective host tegument. The adult worms are blood flukes that live in the mesenteric veins (*S. mansoni* and *S. japonicum*), the rectal veins (*S. intercalatum* and* S. guineensis*), or the vesical veins (*S. haematobium*). The major chronic health problems associated with schistosomiasis are mainly caused by eggs that are not removed from the body in feces or urine (depending on the schistosome location). Regarding the three last species, *S. haematobium, S. guineensis, *and* S. intercalatum,* more specifically, a review of their geographical distribution shows that while *S. haematobium* is distributed largely through Africa and Middle East, *S. guineensis* is known to have a historical restricted geographical range encompassing the Lower Guinea zone, including Cameroon, Equatorial Guinea, Gabon, and Sao Tomé [5–10], and *S. intercalatum* is strictly limited to the Democratic Republic of the Congo [5]; therefore, all the papers referring to *S. intercalatum* from areas out of the Democratic Republic of the Congo will be considered as referring to *S. guineensis*. However, it has been shown that *S. guineensis* is expanding its geographical distribution beyond its historical range, especially to West Africa. This includes Nigeria, where the low prevalence observed suggests a recent introduction of this species [11, 12], the Dogon Plateau in the Mopti region of Mali, where tourists [13, 14] but not the local community [15] were found to be positive, and Burkina Faso, where one case has been diagnosed [16].

The geographical situation of the Republic of Benin, in between Nigeria, Mali, and Burkina Faso, raises the question about the possible presence of *S. haematobium/S. guineensis* hybrids in this country. Until now, *S. haematobium* was found to be largely distributed in the different departments of this country, while *S. guineensis* was never reported [17]. In this study, we used the morphology of eggs, the high-resolution DNA melting analysis (HRM), and gene sequencing of larvae in order to detect natural interactions between *S. haematobium *and *S. guineensis *in the Republic of Benin.

## 2. Methods

### 2.1. Ethics Statement

This study is part of a larger investigation of schistosomiasis epidemiology, transmission, and control in Benin which has been already partially published [18]. More specifically, the sample gathering conducted for this study was listed as one of the planned activities in the approved protocol of the larger study (CORUS 2 project N° 6069 of the French Ministry of Foreign Affairs between the Faculté de la Santé (Abomey-Calavi University, Benin) and the Laboratoire Ecologie et Evolution des Interactions (Perpignan, France), which is a WHO collaborating center (FRA-69). Since the experimental work was made on urine and faeces and did not carry a risk of harm on the human subjects and that no experimental procedures of any kind were conducted on the study participants, the Ethical Committee was not asked to approve these experiments. However, local health authorities were informed about the purpose and procedures of the study. All procedures included in the study participation are part of good routine management for schistosomiasis diagnosis in the country. It was considered sufficient and appropriate by the local investigators to obtain verbal informed consent, in lieu of written consent, since the participation in the study only consisted of testing of urine and stool specimens and did not consist of harmful sampling. Participants were informed about their involvement. All participants, their parents/guardian, and teachers, gave verbal consent for urine and stool examination. Information to the participants and oral consent were witnessed by the director of the school and the teachers and the name of the participant was then registered by the research team. All consent process was documented in a logbook. The data were analyzed anonymously. At the end of the study, all individuals were invited to learn about their parasitological results and free treatment was offered to participants found to be positive for schistosomiasis (single 40 mg/kg dose of Praziquantel).

### 2.2. Recovery of Eggs and Miracidia

**Figure 1:**
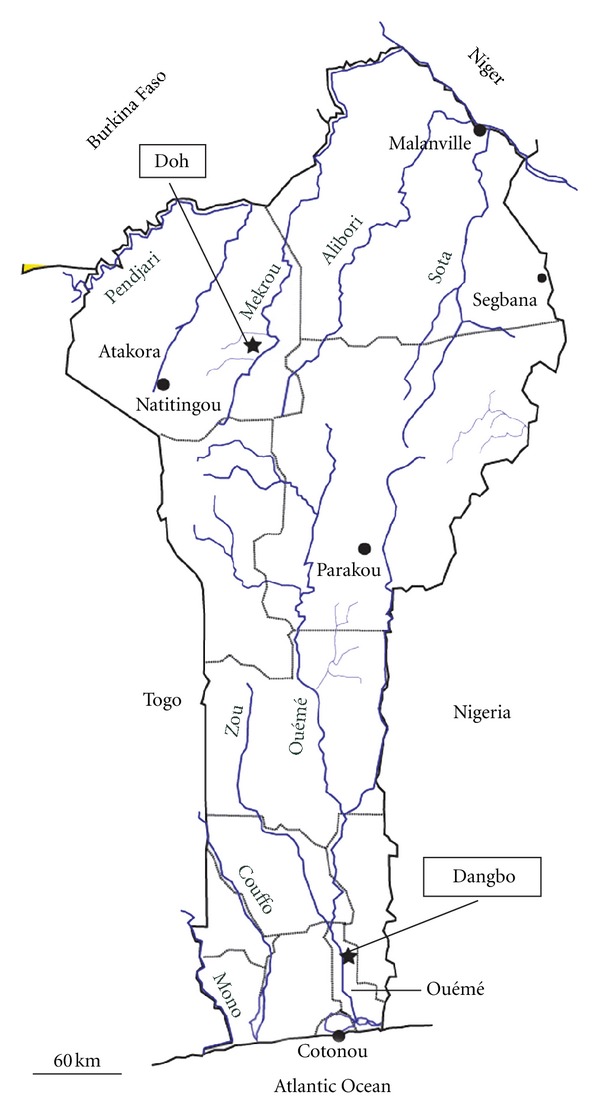
Locations of the study sites of Doh (north Benin) and Dangbo (south Benin).

The study was carried out at schools in two villages in December 2010: (i) at Doh school in the rural village of Doh, Atacora Department, north Benin (1°  56′  43.33′′ E; 10°  20′  17.49′′ N; Figure 1), and (ii) at Dogla school, the village of Dangbo, Ouémé Department near Porto-Novo (the capital of Benin), 20 km from the Nigerian border (2°  32′  53.3′′ E; 6°  35′  51.2′′ N; Figure 1). Urine samples were collected from 92 children at the Doh school (39 girls, 53 boys; 9–14 years of age) and 74 school children at the school in Dangbo (30 girls, 44 boys; 8–15 years of age). In the laboratory, the volume of each urine sample was measured, each sample was then individually passed through a 45 *μ*m pore size sieve (9‰  NaCl saline solution was used to wash the sample through the filter) and any eggs trapped on the sieve were collected in a beaker containing 20 mL saline solution. The total number of eggs was counted from three aliquots of 1 mL which permitted to know the mean number of eggs per mL which then allowed us to calculate the total number of eggs per 10 mL of urine. Seven of the 92 children from the Doh school (7.6%; 2 girls and 5 boys) were positive for the presence of eggs, and the number of eggs per 10 mL of urine ranged from 1.7 to 514. Two of the 74 children from the school in Dangbo (2.7%; 1 girl and 1 boy) were positive for the presence of eggs, and the number of eggs per 10 mL of urine was 3.8 and 320. All the positive children were asked to provide a stool sample and a second urine sample for the further morphological and molecular analyses. In the laboratory, the stool sample of each child was passed through a series of sieves (425 *μ*m, 180 *μ*m, 106 *μ*m and 45 *μ*m pore size), washed through with 9‰  NaCl saline solution, and the residue retained on the 45 *μ*m pore-size sieve was collected in a beaker containing saline solution. No schistosome eggs were found in any of the fecal samples from either school. The urine samples from the positive children from the Doh school were combined and passed through a 45 *μ*m pore size sieve, washing through with saline, and the eggs trapped on the sieve were collected in a beaker containing saline solution. The same procedure was followed for the positive urine samples from the school at Dangbo. A subsample of the eggs was used immediately in saline solution for morphological analyses. The remaining eggs were left to hatch in spring water and each of the resulting miracidia was transferred using a Pasteur pipette into a 1.5 mL PCR reaction tube in 2 *μ*L of well water for molecular analysis. We verified the actual presence of the miracidium and also that there was only one miracidium in each tube, using a binocular microscope.

### 2.3. Egg Morphometry

**Figure 2:**
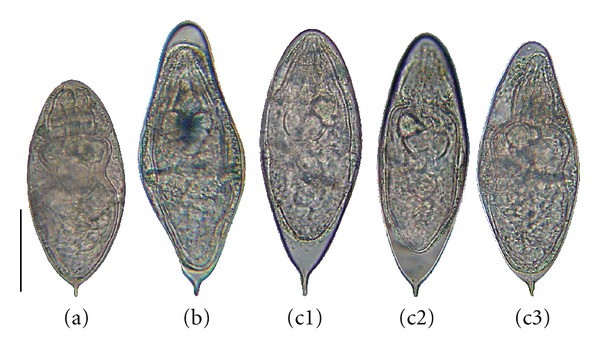
Egg morphology. Typical morphologies of eggs of the *S. haematobium* morphotype (a), the *S. guineensis* morphotype (b), and the morphotype intermediate between *S. haematobium* and *S. guineensis* (c). Bar represents 50 *μ*m.

Eggs from each locality (100 from Doh and 128 from Dangbo) were chosen at random in groups of 5–10 and mounted in saline solution (9‰  NaCl) beneath cover-slips on glass slides using a Pasteur pipette. Only eggs containing a living miracidium were used. The total length (including the terminal spine) and the maximum width of each egg were measured by microscopy. The eggs were grouped according to shape: the *S. haematobium *morphotype included round eggs (Figure 2(a)), the *S. guineensis *morphotype included lozengic eggs (Figure 2(b)), and the intermediate morphotype included eggs whose shape was elongate and intermediate between those of *S. haematobium* and *S. guineensis* (Figures 2(c1), 2(c2), and 2(c3)).

### 2.4. Molecular Analyses

#### 2.4.1. DNA Extraction and Primers Used for PCR Analyses

DNA was extracted from 96 miracidia from Doh and 104 from Dangbo, according to the protocol of [19], and was stored at room temperature. Five adult male worms from Niger (Africa) of known identity (*S. haematobium*) and 5 adult male worms from Cameroon (Africa) of known identity (*S. guineensis*) were used as positive controls. DNA from these worms was extracted using the EZNA Tissue DNA Kit (Omega Bio-tek, USA) according to the manufacture's protocol. PCR amplification of the internal transcribed spacer-2 (ITS2) rDNA was performed using the forward primer, 5′-GCATATCAACGCGGG-3′, and the reverse primer, 5′-ACAAACCGTAGACCGAACC-3′ [20]. Cytochrome C oxidase (CO1) mtDNA amplification was performed by PCR using the forward primer, Cox1_schist F: 5′-TCTTTRGATCATAAGCG-3′, and the reverse primer, Cox1_schist-R: 5′-TAATGCATMGGAAAAAAACA-3′ [21].

#### 2.4.2. ITS2 rDNA HRM PCR

HRM is a PCR-based method for detecting DNA sequence variation, which enables detection of both homozygous and heterozygous sequences [22]. Duplex melting is monitored using an intercalating dye that binds to double-stranded DNA, but not to single-stranded DNA. Decreasing fluorescence of the intercalating dye occurs during the process of dissociation of double-stranded DNA during heating. The shape of the melting curve and the temperature of dissociation (peak) depend on the GC content and on the length and sequence of the amplicon. Single nucleotide polymorphisms and other mutations can be detected immediately without sequencing. This method is rapid, highly sensitive, specific, and powerful. PCR was performed using real-time PCR (LightCycler 480 Instrument II) in a total reaction volume of 10 *μ*L comprising 2 *μ*L of DNA diluted 10-fold for the miracidia [19] and 30 ng of DNA for the adult worms, 1 × Master Mix (high resolution melting dye; Roche Diagnostics, Mannheim, Germany), 0.3 *μ*M forward primer, 0.3 *μ*M reverse primer and 3 mM MgCl_2_. The reaction conditions for both miracidia and adults included an activation step at 95°C for 10 min followed by 50 cycles of 95°C for 15 s, 65°C for 15 s, and 72°C for 20 s. Prior to the HRM step the products were heated to 95°C for 1 min and to 40°C for 1 min. HRM was carried out over the temperature range 65–95°C, rising at 0.02°C per second, with 25 acquisitions per degree. All reactions were performed in 96-well microtiter plates. HRM analysis was performed using the LightCycler 480 software release 1.5.0.

#### 2.4.3. CO1 mtDNA PCR

PCR was performed in a total reaction volume of 30 *μ*L comprising 2 *μ*L of DNA diluted 10-fold for the miracidia [19] and 30 ng of DNA for the adult worms, 1 × colorless GoTaq flexi buffer (Promega, Madison, WI, USA), 1.5 mM MgCl_2_ (Promega, Madison, WI, USA), 0.2 mM of each dNTP (Promega, Madison, WI, USA), 0.5 *μ*M forward primer, 0.5 *μ*M reverse primer and 1U GoTaq Hot Start Polymerase (Promega, Madison, WI, USA). The reaction conditions for both miracidia and adults included an activation step of 95°C for 2 min 30 s, followed by 40 cycles of 95°C for 30 s, 48°C for 40 s, and 72°C for 1 min 10 s, and a final extension at 72°C for 5 min.

#### 2.4.4. Partial ITS2 Sequencing (207 bp)

Partial ITS2 of a subset of the samples was sequenced (GATC Biotech; Konstanz, Germany) using the same forward and reverse primers as for the amplification by PCR. The sequences were manually edited using Sequencher 4.5 (Gene Codes Corp., Ann Arbor, MI, USA) and were aligned using the BioEdit sequence editor software version 7.0.5.3.

#### 2.4.5. Partial CO1 Sequencing (898 bp)

Partial CO1 of a subset of the samples (including all the* S. guineensis* ITS2 genotypes highlighted by the ITS2 rDNA HRM PCR) and of the controls was sequenced (GATC Biotech; Konstanz, Germany) using the reverse primer. The sequences were manually edited using Sequencher 4.5 (Gene Codes Corp.) and were aligned using the BioEdit sequence editor software version 7.0.5.3.

### 2.5. Statistical Analyses

Pairwise comparisons of the proportions of eggs of each morphotype and genotype were made between both localities using the *Z-*test and OpenStat software. Egg sizes were compared using the Mann-Whitney (U), Kruskal-Wallis (KW) and Dunn tests, and the GraphPad Instat software, version 3.05. Means are presented with the standard error. Statistical significance was assessed at the *P* < 0.05 level.

## 3. Results

### 3.1. Egg Morphometry

Three egg morphotypes were detected in samples from the two study localities. In Doh samples, the proportions of eggs of the *S. haematobium*, intermediate and *S. guineensis* morphotypes were 25, 58, and 17%, respectively, while in the Dangbo samples the proportions were 74, 22, and 4%, respectively. The proportion of the *S. haematobium* morphotype was significantly higher in Dangbo samples (Z-stat = −7.4;  *P* < 0.0001), while the proportions of the intermediate and *S. guineensis* morphotypes were significantly higher in the Doh samples (Z-stat = 5.6, *P* < 0.0001 for the intermediate morphotype; Z-stat = 3.3, *P* = 0.0009 for the *S. guineensis* morphotype).

The mean length and mean width of the three morphotypes in the Doh and Dangbo samples are shown in Table 1. At each locality the length of the eggs was shortest for the *S. haematobium* morphotype, intermediate for the intermediate morphotype, and longest for the *S. guineensis* morphotype. The differences were significant between the three morphotypes (KW-stat = 25.2, *P* < 0.0001, and post hoc Dunn test *P* < 0.01 for Doh samples; KW-stat = 7.8, *P* < 0.02, and post hoc Dunn test *P* > 0.05 for Dangbo samples). No significant difference was found among the three morphotypes with respect to the width (KW-test,  *P* > 0.05). Comparison of egg sizes between the localities for each morphotype showed only one significant difference, associated with the *S. haematobium* morphotype, where the length was significantly greater in Dangbo samples relative to Doh samples (U-stat = 826, *P* = 0.02).

### 3.2. High Resolution Melting Analysis of the ITS2 rDNA

**Figure 3:**
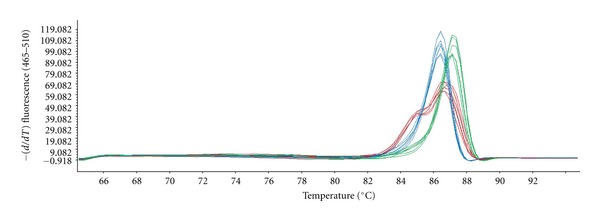
Results of the high resolution melting analysis. Melting peaks of 6 randomly selected individuals from the *S. haematobium *(green), hybrid (red), and *S. guineensis* (blue) patterns.

The HRM curves showed polymorphism within our miracidial samples. The LightScanner software identified three groups of curves (Figure 3) with homogeneous intragroup shapes and homogeneous temperatures of dissociation: (i) those with a typical homozygous melting pattern of only one peak and a high temperature of dissociation, which correspond to *S. haematobium*; all of the 5 *S. haematobium* controls harbored this pattern; (ii) those with a typical homozygous melting pattern with only one peak and a low temperature of dissociation, which correspond to *S. guineensis*; all of the 5 *S. guineensis* controls harbored this pattern; (iii) those with a characteristic heterozygous pattern of two peaks and with intermediate temperature of dissociation, which correspond to the* S. haematobium/S. guineensis* hybrids. 

The proportions of miracidia of the *S. haematobium*, hybrid, and *S. guineensis* ITS2 genotypes were 26, 51, and 23%, respectively, among the Doh samples, and 72, 23, and 5%, respectively, among samples from the Dangbo locality. Comparative analysis showed that the proportion of the *S. haematobium* genotype was significantly higher in the Dangbo samples compared to the Doh samples (Z-stat = −6.5,  *P* < 0.0001) but the proportions of the *S. guineensis* and the hybrid genotypes were significantly higher in the Doh samples compared to the Dangbo samples (Z-stat = 4.1, *P* < 0.0001 for the hybrid genotype, and Z-stat = 3.7, *P* = 0.0002 for the *S. guineensis* genotype).

### 3.3. (ITS2) rDNA Sequencing

**Figure 4:**
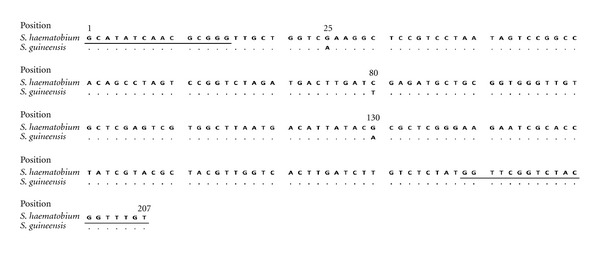
ITS2 fragment sequences showing the three single-nucleotide polymorphisms between *S. haematobium* and *S. guineensis*. The priming sites are underlined.

There are 3 known polymorphic positions between the two species *S. haematobium* and *S. guineensis*. From the 5′ end of the fragment these occur at position 25, where G (in *S. haematobium*) is replaced by A (in *S. guineensis*); at position 80, where C (in *S. haematobium*) is replaced by T (in *S. guineensis*); and at position 130, where G (in *S. haematobium*) is replaced by A (in *S. guineensis*). At all other positions, the sequences are identical (Figure 4). Detection of hybrids is made by checking for the presence of double peaks at these 3 polymorphic positions. The ITS2 sequence analysis conducted in this study confirmed the assignments based on the HRM analysis. The *S. haematobium* HRM ITS2 genotypes produced single-chromatogram peaks and specific SNPs (G, C, and G) at the three polymorphic positions, as for the species *S. haematobium*. The *S. guineensis* HRM ITS2 genotypes produced single-chromatogram peaks and had the specific SNPs A, T, and A at the three polymorphic positions, as for the species *S. guineensis*. The hybrid HRM ITS2 genotypes produced double-chromatogram peaks and had the SNPs G and A, C and T, and G and T at the three polymorphic positions.

### 3.4. (CO1) mtDNA Sequencing

CO1 mtDNA sequencing showed substitutions at 59 of the 898 sites between *S. haematobium* from Niger and *S. guineensis *from Cameroon (used as controls). This corresponds to a 6.6% bp difference between the two species. Irrespective of the HRM ITS2 genotype (*S. haematobium*, hybrid or even *S. guineensis*), the CO1 sequences of the Benin individuals were very similar to that of *S. haematobium* from Niger; substitutions relative to the Niger control occurred at 6 to 8 of the total 898 sites (0.7 to 0.9% bp difference), while the percentage bp difference of the Benin individuals from *S. guineensis* from Cameroon varied from 5.7 to 5.9%.

## 4. Discussion

The morphological results showed three egg morphotypes* S. haematobium*, *intermediate, and S. guineensis*. Comparison of egg sizes found in the present study with published data shows that: (i) the mean egg length of the *S. haematobium* morphotype (146 *μ*m for Dangbo samples and 139 *μ*m for Doh samples) was similar to that of *S. haematobium* reported by Pitchford [23] (131–142 *μ*m), Loker [24] (144 *μ*m), and Richard-Lenoble and colleagues [25] (146 *μ*m); (ii) the mean egg length of the intermediate morphotype (151 *μ*m for both Dangbo and Doh samples) was similar to that of hybrids collected from urine by Mintsa Nguema and colleagues [26] (144 and 159 *μ*m) and Brown and colleagues [8] (151 *μ*m); (iii) the mean egg length of the *S. guineensis* morphotype (158 *μ*m for Dangbo samples and 164 *μ*m for Doh samples) was less than that of *S. guineensis* collected from feces by Wright and colleagues [6] (172 *μ*m), Richard-Lenoble and colleagues [25] (173 *μ*m), Southgate and colleagues [7] (175–189 *μ*m), and Pagès and Théron [27] (188 *μ*m). Consequently, our morphological data are correlated with previous data for *S. haematobium* and hybrids of *S. haematobium* and *S. guineensis*, but inconsistent with previous data for *S. guineensis*. The absence of a correlation between our *S. guineensis* morphotype and the other reports for *S. guineensis* may result in the urine-only origin of our *S. guineensis* morphotype; however, it is supported by the fact that no schistosome eggs were found in the feces of the individuals whose urine was found positive for the presence of schistosome eggs; indeed, the egg laying site of *S. guineensis *is the rectal veins, and the eggs of this species are recovered in the feces and not in the urine.

Three genotypes could be detected by the ITS2 HRM analysis in our study* S. haematobium*, *hybrid, and S. guineensis*. The hybrid genotype comprised 51% and 23% of the total genotypes at Doh and Dangbo, respectively, and the *S. guineensis* genotype comprised 23% and 5% of the genotypes at these sites, respectively. These data were consistent with the morphological analysis for each locality, and for each morphogenotype (*Z-*test; *P* > 0.05). According to the HRM results only, we could conclude that* S. haematobium, S. guineensis *and their hybrids are present in the Republic of Benin. Hybrids between *S. haematobium *and *S. guineensis *were found to exist only in Cameroon [28, 29]. However, the CO1 gene sequencing analysis showed that the maternal lineages of our samples were identified as *S. haematobium*, even of all of those that were *S. guineensis* ITS2 genotyped. Thus, in both of our studied areas, *S. haematobium *genome has been introgressed by *S. guineensis *genes. *S. haematobium* is known to interact with other species of the terminal-spined egg group, as *S. guineensis* in its historical geographical zone [30] or *S. bovis*, agent of a bovine schistosomiasis [31, 32].

The history of the introgression observed in the Republic of Benin is difficult to explain by an initial cross between a male *S. haematobium *and a female* S. guineensis*, as no trace of* S. guineensis *could be detected in the mtDNA. The introgressed *S. haematobium* should have been the result of an initial cross between a male *S. guineensis* and a female *S. haematobium*, but this hypothesis seems unlikely because of the known inability of *S. guineensis* males to compete effectively with *S. haematobium* males [33], and because our samples were collected from urine, which does not reflect the egg laying site of *S. guineensis*. However, this hypothesis becomes likely if *S. haematobium* females were active in the choice of the egg laying site. A recent study on female preference for genetically dissimilar males [34] highlights that the role of schistosome females in male-female interactions in the vertebrate host has probably been underestimated.

Further field work is needed in order to know to what extent introgression of *S. guineensis* genes in *S. haematobium* is present in West Africa, especially where *B. forskalii* has been found in natural conditions; a recent review on human schistosomiasis in the Economic Community of West African States (ECOWAS) showed that *B. forskalii* is present in 11 among the 15 countries of ECOWAS (73.3%); these countries are: Mali, Niger, Burkina Faso, Cape Verde, Senegal, The Gambia, Sierra Leone, Côte d'Ivoire, Togo, Benin, and Nigeria [17]. Such field studies exploring introgression of *S. guineensis* genes in *S. haematobium* are needed since they have at least two large potential public health impacts. The first impact is that hybrids in schistosomes are known to present heterosis (higher fecundity, faster maturation time, wider intermediate host spectrum) [35–37] which may have an impact on the evolutionary process prevailing in the hybrid geographical zones [38]. Furthermore, hybridization may also lead to complications in the control of the disease as chemotherapy resistance; for example, Pitchford and Lewis [39] have suggested that the poor response of *S. mattheei* to antihelminthic treatment may be due to hybridization with *S. haematobium*. Further field work is also needed in order to search for the presence of *S. guineensis* in Bénin since, in this paper, only the individuals that released schistosome eggs in the urine were further diagnosed for eggs in fecal samples.

## Figures and Tables

**Table 1:** Egg morphometry.

Locality	Doh			Dangbo		
Morphotype	*S. haematobium*	Intermediate	*S. guineensis*	*S. haematobium*	Intermediate	*S. guineensis*

Length (*μ*m)	139 ± 3	151 ± 2	164 ± 2	146 ± 1	151 ± 3	158 ± 7
Width (*μ*m)	62 ± 1	59 ± 1	59 ± 2	61 ± 1	59 ± 1	61 ± 3

Mean ± standard error.
